# Supplementary material for: Analyzing real-world adverse events of spironolactone with the FAERS database
Source: PLoS One. 2025 Sep 17;20(9):e0330659. doi: 10.1371/journal.pone.0330659 (PMC12443299; doi:10.1371/journal.pone.0330659)
Supplement: S1 Table — (DOCX) [file pone.0330659.s001.docx]

**S1 Table. Application Example of Signal Detection Methods for "Endometriosis Male".**

| Method | Formula | ﻿Threshold | 2×2 Contingency Table Parameters (Example Assumptions) | Calculation Results | Meets Threshold |
| --- | --- | --- | --- | --- | --- |
| ROR | $ROR=\frac{a / c}{b / d}$ | a ≥ 3  ROR ≥ 3  ﻿95%CI (lower limit) > 1 | (a=7) (spironolactone-related ADEs)  (b=10) (spironolactone use in non-ADEs) | ROR=13615.84  95% CI: (1675.1, 110674.81) | Yes |
|  | $SE(lnROR)=\sqrt{\frac{1}{a}+\frac{1}{b}+\frac{1}{c}+\frac{1}{d}}$ |  | (c=0.005) (non-spironolactone ADEs)  (d=10000) (non-spironolactone, non-ADEs) |  |  |
|  | $95\%CI= e^{\ln\left( \mathrm{ROR} \right)\pm1.96se}$ |  |  |  |  |
| PRR | $PRR=\frac{a / (a+b)}{c / (c+d)}$ | a ≥ 3  PRR ≥ 2  ﻿95%CI (lower limit) > 1 | Same as above | PRR=13612.3  95%CI: (1671.59, 110849.54) | Yes |
|  | $SE(lnPRR)=\sqrt{\frac{1}{a}-\frac{1}{a+b}+\frac{1}{c}-\frac{1}{c+d}}$ |  |  |  |  |
|  | $95\%CI= e^{\ln\left( \mathrm{PRR} \right)\pm1.96se}$ |  |  |  |  |
| BCPNN | $IC=\log_{2}\frac{p(x, y)}{p\left( x \right)p(y)}= \log_{2}\frac{a(a+b+c+d)}{(a+b)(a+c)}$ | IC025>0 | Same as above | IC=10.73  IC025=9.36 | Yes |
|  | $E(IC)=\log_{2}\frac{(a+\gamma11)(a+b+c+d+\alpha)(a+b+c+d+\beta)}{\left( a+b+c+d+\gamma\right)(a+b+\alpha1)(a+c+\beta1)}$ |  |  |  |  |
|  | $V\left( \mathrm{IC} \right)=\frac{1}{{(ln2)}^{2}}[\frac{\left( a+b+c+d \right)-a+\gamma-\gamma11}{\left( a+\gamma11 \right)\left( 1+a+b+c+d+\gamma\right)}+\frac{\left( a+b+c+d \right)-\left( a+b \right)+a-\alpha1}{\left( a+b+\alpha1 \right)\left( 1+a+b+c+d+\alpha\right)}+\frac{\left( a+b+c+d+\alpha\right)-\left( a+c \right)+\beta-\beta1}{\left( a+b+\beta1 \right)\left( 1+a+b+c+d+\beta\right)}]$ |  |  |  |  |
|  | $\gamma=\gamma11\frac{(a+b+c+d+\alpha)(a+b+c+d+\beta)}{\left( a+b+\alpha1 \right)(a+c+\beta1)}$ |  |  |  |  |
|  | $IC-2SD=E\left( \mathrm{IC} \right)-2 \sqrt{V(IC)}$ |  |  |  |  |
| EBGM | $EBGM=\frac{a(a+b+c+d)}{\left( a+c \right)(a+b)}$ | EBGM05>2 | Same as above | EBGM=1702.41  EBGM05=294.87 | Yes |
|  | $SE(lnEBGM)=\sqrt{\frac{1}{a}+\frac{1}{b}+\frac{1}{c}+\frac{1}{d}}$ |  |  |  |  |
|  | $95\%CI= e^{\ln\left( \mathrm{EBGM} \right)\pm1.96se}$ |  |  |  |  |
