# Supplementary material for: Analyzing real-world adverse events of spironolactone with the FAERS database
Source: PLoS One. 2025 Sep 17;20(9):e0330659. doi: 10.1371/journal.pone.0330659 (PMC12443299; doi:10.1371/journal.pone.0330659)
Supplement: Attachment — Submitted filename: Response to Reviewers.docx [file pone.0330659.s003.docx]

Dear Editors and Reviewers:

On behalf of my co-authors, we thank you very much for giving us an opportunity to revise our manuscript, and we appreciate the editor and reviewers very much for their positive and constructive comments and suggestions on our manuscript entitled “Analyzing Real-world Adverse Events of Spironolactone with the FAERS Database”. Those comments are all valuable and very helpful for revising and improving our paper, as well as the important guiding significance to our research. We have studied comments carefully and have made corrections which we hope will meet with approval. The main corrections in the paper and the responses to the reviewers’ comments are as follows:

Responds to the reviewers’ comments:

Journal Requirements:

When submitting your revision, we need you to address these additional requirements.

**1) Please ensure that your manuscript meets PLOS ONE's style requirements, including those for file naming. The PLOS ONE style templates can be found at**

**https://journals.plos.org/plosone/s/file?id=wjVg/PLOSOne_formatting_sample_main_body.pdf and**

**https://journals.plos.org/plosone/s/file?id=ba62/PLOSOne_formatting_sample_title_authors_affiliations.pdf**

***Response:*** We have carefully revised the manuscript to fully comply with PLOS ONE's style requirements, including proper file naming conventions as specified in the provided templates. All structural elements (such as section headings, figure/table formats, and reference citations) have been formatted according to the journal's guidelines, and supporting information files have been named appropriately. The document now adheres to the specified styling for titles, authors, affiliations, and supplementary materials, ensuring alignment with PLOS ONE's submission standards.

**2) We note that Figure 3 in your submission contain map images which may be copyrighted. All PLOS content is published under the Creative Commons Attribution License (CC BY 4.0), which means that the manuscript, images, and Supporting Information files will be freely available online, and any third party is permitted to access, download, copy, distribute, and use these materials in any way, even commercially, with proper attribution. For these reasons, we cannot publish previously copyrighted maps or satellite images created using proprietary data, such as Google software (Google Maps, Street View, and Earth). For more information, see our copyright guidelines: http://journals.plos.org/plosone/s/licenses-and-copyright.We require you to either (1) present written permission from the copyright holder to publish these figures specifically under the CC BY 4.0 license, or (2) remove the figures from your submission: You may seek permission from the original copyright holder of Figure 3 to publish the content specifically under the CC BY 4.0 license. We recommend that you contact the original copyright holder with the Content Permission Form (http://journals.plos.org/plosone/s/file?id=7c09/content-permission-form.pdf) and the following text: “I request permission for the open-access journal PLOS ONE to publish XXX under the Creative Commons Attribution License (CCAL) CC BY 4.0 (http://creativecommons.org/licenses/by/4.0/). Please be aware that this license allows unrestricted use and distribution, even commercially, by third parties. Please reply and provide explicit written permission to publish XXX under a CC BY license and complete the attached form.” Please upload the completed Content Permission Form or other proof of granted permissions as an ""Other"" file with your submission. In the figure caption of the copyrighted figure, please include the following text: “Reprinted from [ref] under a CC BY license, with permission from [name of publisher], original copyright [original copyright year].”**

***Response:*** Thank you for bringing this to our attention. We have promptly removed Figure 3 from the manuscript as requested, and confirm that this modification does not impact the scientific integrity or narrative flow of the original content. All references to the figure within the text have been carefully adjusted to maintain coherence. We appreciate your guidance in ensuring compliance with PLOS ONE's licensing and copyright standards, and stand ready to provide any further documentation or revisions as needed.

**3) Please remove your figures from within your manuscript file, leaving only the individual TIFF/EPS image files, uploaded separately. These will be automatically included in the reviewers’ PDF.**

***Response:*** We have followed your instructions to remove all figures from the main manuscript file. The figures have been saved as individual TIFF/EPS image files and will be uploaded separately as required. This ensures they will be automatically included in the reviewers’ PDF, while the main document now only contains text and figure citations for clarity.

**4) Please include captions for your Supporting Information files at the end of your manuscript, and update any in-text citations to match accordingly. Please see our Supporting Information guidelines for more information:** [**http://journals.plos.org/plosone/s/supporting-information**](http://journals.plos.org/plosone/s/supporting-information)**.**

***Response:*** We have added captions for the Supporting Information files at the end of the manuscript and updated the in-text citations accordingly. In the Methods section, the corresponding in-text citation has been revised to "S1 Table" to match this caption.

**Reviewer 1**

Lin et al entitle "Analyzing Real-world Adverse Events of Spironolactone with the FAERS Database" clearly reported that Spironolactone could be adverse drug for heart failure, hypertension, and hyperaldosteronism.

**1) Why are you using the FAERS database to study this drug, it should be that someone else has done this with this similar database similar methodology, so you can write cite in the INTRODUCTION section about some specific other similar studies, such as recommending a few (It is equivalent to saying that someone else has done this type of research using the FAERS database, and you can use this database to do research related to Spironolactone as well):【1】Wang Y, Zhao B, Yang H, Wan Z. A real-world pharmacovigilance study of FDA adverse event reporting system events for sildenafil. Andrology. 2024 May;12(4):785-792. doi: 10.1111/andr.13533. Epub 2023 Sep 19. PMID: 37724699.【2】Zhao B, Fu Y, Cui S, Chen X, Liu S, Luo L. A real-world disproportionality analysis of Everolimus: data mining of the public version of FDA adverse event reporting system. Front Pharmacol. 2024 Mar 12;15:1333662. doi: 10.3389/fphar.2024.1333662. PMID: 38533254; PMCID: PMC10964017.【3】Yang H, Wan Z, Chen M, Zhang X, Cui W, Zhao B. A real-world data analysis of topotecan in the FDA Adverse Event Reporting System (FAERS) database. Expert Opin Drug Metab Toxicol. 2023 Apr;19(4):217-223. doi: 10.1080/17425255.2023.2219390. Epub 2023 May 30. PMID: 37243615.【4】Zhao B, Zhang X, Chen M, Wang Y. A real-world data analysis of acetylsalicylic acid in FDA Adverse Event Reporting System (FAERS) database. Expert Opin Drug Metab Toxicol. 2023 Jan-Jun;19(6):381-387. doi: 10.1080/17425255.2023.2235267. Epub 2023 Jul 12. PMID: 37421631.【5】Li, Jie, Zhao, Bin, Zhu, YongQing, Wu, Jibiao, Vitreoretinal Traction Syndrome, Nitrituria and Human Epidermal Growth Factor Receptor Negative Might Occur in the Aromatase-Inhibitor Anastrozole Treatment, International Journal of Clinical Practice, 2024, 5132916, 9 pages, 2024. https://doi.org/10.1155/2024/5132916【6】Zhong, C., Zheng, Q., Zhao, B., & Ren, T. (2024). A real-world pharmacovigilance study using disproportionality analysis of United States Food and Drug Administration Adverse Event Reporting System events for vinca alkaloids: comparing vinorelbine and Vincristine. Expert Opinion on Drug Safety, 23(11), 1427–1437. https://doi.org/10.1080/14740338.2024.2410436.**

***Response:*** Thank you for the feedback. We have incorporated the suggested references in the Introduction section to contextualize the use of the FAERS database, aligning with prior studies that have employed similar methodologies. This addition strengthens the rationale for our approach and highlights the database's utility in pharmacovigilance research. These revisions have been incorporated into the revised **Introduction section (paragraph 3)**.

**2) The manuscript mentions the use of ROR, PRR, BCPNN, and EBGM for signal detection. However, the thresholds for significance (e.g., ROR ≥3, PRR ≥2) are not explicitly justified in the context of this study. Please briefly cite relevant literature or guidelines supporting these thresholds to enhance methodological transparency.**

***Response:*** Thank you for pointing out the need to justify the thresholds for signal detection methods. We have addressed this by supplementing the methodology with citations to relevant literature. The thresholds were selected based on established standards in pharmacovigilance: ROR ≥ 3 and 95% CI lower limit > 1 follow Rothman et al. (2004), PRR ≥ 2 aligns with Evans et al. (2001), BCPNN uses IC025 > 0 as recommended by Bate et al. (1998), and EBGM05 > 2 follows Dumouchel (1999). These references now appear in the manuscript to validate the criteria, ensuring methodological transparency and consistency with prior research in the field. These revisions have been incorporated into the revised **Methods section 2.2 (paragraph 2)**.

**3) The Data Availability section states, "The dataset [...] is available from the corresponding author upon request," but the response to the FAERS data availability question on page 5 states, "All relevant data are within the manuscript and its Supporting Information files." Clarify this discrepancy and ensure alignment with PLOS ONE’s data policy (e.g., specify whether raw FAERS data are publicly accessible or require request).**

***Response:*** Thank you for pointing out the discrepancy in the data availability statements. The confusion stems from the distinction between the raw FAERS data and the study-specific processed dataset. The raw FAERS data used in this analysis are publicly accessible via the FDA’s official repository (https://fis.fda.gov/extensions/FPD-QDE-FAERS/FPD-QDE-FAERS.html), as acknowledged in the manuscript. In contrast, the processed dataset (e.g., cleaned reports, annotated variables, and analysis outputs) generated during this study is available from the corresponding author upon reasonable request, as stated in the Data Availability section. To align with PLOS ONE’s data policy, the statement has been revised to clarify: "The raw FAERS data used in this study are publicly available from the U.S. Food and Drug Administration (FDA) (https://fis.fda.gov/extensions/FPD-QDE-FAERS/FPD-QDE-FAERS.html). The processed dataset and analysis scripts generated during the current study are available from the corresponding author upon reasonable request." This revision explicitly differentiates between publicly accessible raw data and study-generated processed data, ensuring transparency and compliance with journal guidelines. These revisions have been incorporated into the revised **Availability of data and materials section**.

**4) Figures 1-3 and Tables 1-5 are referenced in the text but lack captions in the submitted manuscript (e.g., "Fig 1: The flow diagram..." appears only in the text layer). Ensure all figures and tables include descriptive captions in the main manuscript file for clarity.**

***Response:*** Thank you for noting the absence of captions for Figures 1-3 and Tables 1-5 in the manuscript. We have addressed this by incorporating descriptive captions for all figures and tables directly within the main document to enhance clarity and compliance with publishing standards. Each figure caption now concisely outlines the workflow or key elements. These revisions ensure that visual and tabular content is self-explanatory and fully integrated with the text, eliminating reliance on in-text descriptions alone and aligning with academic formatting requirements.

**5) While rare ADEs like "endometriosis male" (n=7) are highlighted, the discussion could benefit from addressing potential confounding factors (e.g., comorbidities, concomitant medications) that might influence these signals. A brief acknowledgment of these limitations would strengthen the interpretation.**

***Response:*** We acknowledge the potential confounding factors influencing rare ADE signals (e.g., male endometriosis, n=7). FAERS data lack detailed documentation of comorbidities (e.g., cirrhosis, hormone therapy history) and concomitant medications (e.g., antiandrogens, diuretics), which may independently contribute to these events or interact with spironolactone. The voluntary reporting nature may introduce selection bias, hindering causal inference. Prospective studies or electronic health record analyses controlling for clinical confounders are needed to validate these signals. We have added relevant content in the discussion section. These revisions have been incorporated into the revised **Discussion section (last paragraph)**.

**6) The ethics statement on page 3 notes that ethical approval was not required. However, the FAERS database includes patient-reported data. Please confirm compliance with FAERS data usage policies and clarify whether patient identifiers were anonymized in accordance with ethical standards.**

***Response:*** Our study adheres to FAERS data usage policies and ethical standards. The FAERS database publicly provides anonymized adverse event reports, with patient identifiers and sensitive information removed in compliance with FDA regulations. As stated in the ethics statement (**Section 12**), the research involved secondary analysis of fully anonymized, publicly available data, which does not constitute human subjects research requiring ethical approval. All data used in this study were de-identified prior to access, aligning with both FAERS guidelines and institutional ethical requirements for non-identifiable dataset analysis.

**7) Some references lack consistent formatting (e.g., missing DOIs, inconsistent journal abbreviations). For example, reference 33 (Greenblatt et al., 1973) does not include a DOI. Ensure all references adhere to PLOS ONE’s formatting guidelines.**

***Response:*** We have standardized all references to comply with PLOS ONE’s formatting guidelines, including adding missing DOIs and ensuring consistent journal abbreviations. For references without DOIs (e.g., older studies), we’ve followed the journal’s requirements for legacy publications.

**8) Terms like "SBMA" (spinal bulbar muscular atrophy) and "DHL" (double-hit lymphoma) are introduced in the Discussion without prior definition. Define abbreviations upon first mention to improve readability for non-specialist readers.**

***Response:*** We appreciate the feedback on enhancing readability for non-specialist readers. In the revised manuscript, we have ensured that abbreviations such as "SBMA" (spinal bulbar muscular atrophy) and "DHL" (double-hit lymphoma) are defined at their first occurrence in the Discussion section. These revisions have been incorporated into the revised **Discussion section (paragraph 5)**.

Reviewer 2:

The study addresses an important and timely issue in pharmacovigilance by leveraging the FAERS database to explore the safety profile of spironolactone, including the detection of rare and potentially novel adverse events. The methodology is robust, and the findings are potentially impactful for clinical and regulatory audiences.

However, there are some issues which are to be clarified:

**1) While you’ve included the key formulas and thresholds used for signal detection, I recommend expanding on how these were applied in practice. For instance, did all four criteria need to be met to consider a signal significant? How were discordant results across methods handled? A worked example or supplementary figure might be helpful to illustrate the decision-making process.**

***Response:*** We appreciate the suggestion to elaborate on the practical application of signal detection methods. In this study, a signal was considered significant only when all four statistical methods (PRR, ROR, BCPNN, EBGM) met their respective threshold criteria (e.g., ROR ≥ 3 with 95% CI lower limit > 1, PRR ≥ 2, IC025 > 0, EBGM05 > 2). This multi-algorithm approach was deliberately implemented to minimize false positives by requiring cross-validation across different statistical frameworks. For discordant results—where one method met criteria but others did not—signals were not prioritized, as the combined use of these algorithms aimed to ensure consistency in detecting genuine associations. To illustrate this process, a representative example is provided: for "endometriosis male" (n=7), all four methods yielded statistically significant values (ROR=13615.84, PRR=13612.3, IC=10.73, EBGM=1702.41), exceeding their respective thresholds, which validated its status as a strong signal. While a detailed worked example was not included in the main text, the **supplementary materials** contain a step-by-step demonstration of how these algorithms were applied to a subset of ADEs, including the construction of 2×2 contingency tables and threshold evaluations. This approach ensures transparency in our signal detection methodology and reinforces the reliability of our findings. These revisions have been incorporated into the revised **Methods section 2.2 (paragraph 2)**.

2) **Some of the newly detected signals (e.g., male endometriosis, 5-alpha-reductase deficiency) are quite rare and biologically complex. Adding a brief subsection on the clinical plausibility and supporting literature (if any) would be helpful.**

***Response:*** Thank you for your valuable suggestion. We acknowledge the importance of addressing the clinical plausibility and supporting literature for the newly identified rare signals, such as male endometriosis and 5-alpha-reductase deficiency. In response, we have added a dedicated subsection in **the Discussion section (Paragraph 4)** to elaborate on these aspects. This addition provides biological rationales and cites relevant case reports/studies to support the observed associations, enhancing the manuscript's scientific rigor and clinical relevance. The revision aims to clarify the potential mechanisms underlying these rare events and emphasize the need for further investigation, as outlined in the updated content.

3) **The description of data cleaning and deduplication could be strengthened by including a brief example—for instance, how duplicate reports were identified using CASEID and ISR, or how multiple versions of the same case were handled. This would improve methodological clarity and reproducibility.**

***Response:*** Thank you for your constructive feedback. We agree that clarifying the data cleaning and deduplication process with specific examples enhances methodological transparency. In the revised manuscript, we have added a paragraph detailing how unique identifiers (CASEID and ISR) were used to identify and remove duplicate reports. Specifically, we now describe how cases with the same CASEID but different ISR numbers (indicating updates) were handled by retaining only the most recent version. This addition improves the reproducibility of our methods and aligns with best practices in pharmacovigilance data analysis. These revisions have been incorporated into the revised **Methods section 2.2 (paragraph 1)**.

**4) Abbreviations are used in tables and flow chart. Please provide the explanation for those abbreviations below the tables/figures.**

***Response:*** Thank you for highlighting the need to clarify abbreviations in tables and figures. We have added explanatory notes for all abbreviations below the respective tables and the flow chart in the revised manuscript. These explanations have been inserted as footnotes beneath each table and the flow chart to enhance readability and ensure transparency.

ADDITIONAL COMMENTS FROM THE EDITOR:

**1)** **Abstract: Why are only ROR values only reported. The ROR values you reported are also very large. An explanation is needed regarding this.**

***Response:*** Thank you for your feedback. The decision to prioritize reporting ROR values in the abstract stems from their demonstrated robustness in estimating risk ratios for rare adverse events. Unlike methods such as PRR, ROR provides more stable estimates with minimal bias, particularly when event counts are low. While ROR is highlighted in the abstract, all signals were cross-validated using PRR, BCPNN, and EBGM (as shown in **Table 5** of the manuscript), ensuring methodological rigor. ROR is a widely recognized metric in pharmacovigilance due to its ability to reflect the strength of associations clearly (e.g., ROR ≥ 3 with 95% CI lower limit > 1), aligning with established practices in FAERS database analyses. The disproportionately large ROR values for rare adverse events (e.g., male endometriosis, 5-alpha-reductase deficiency) primarily stem from their extreme rarity in the FAERS database. For instance, when an event like male endometriosis is reported in only 7 cases with spironolactone (a=7) and scarcely occurs in non-spironolactone reports (c≈0), the ratio (a/c)/(b/d) naturally yields an extremely high ROR. The manuscript’s **Methods section** details the application of all statistical approaches, and the abstract’s focus on ROR balances conciseness with scientific validity, efficiently conveying key findings while maintaining transparency.

**2) General: Do adverse event, adverse effect, adverse reaction, and adverse drug event, adverse drug reaction mean the same thing? If so, I suggest you pick one (e.g., ADR) and use it consistently to avoid potential confusion. Also be consistent when using abbreviations.**

***Response:*** Thank you for your thoughtful feedback on terminological consistency. In response, we appreciate the need to clarify our terminology choice. While "adverse drug reaction (ADR)" denotes a confirmed causal relationship, we have elected to use "adverse drug event (ADE)" throughout the manuscript. ADE is defined as any untoward medical occurrence associated with drug use, regardless of established causality, which aligns with the FAERS database’s reporting framework and our study’s retrospective pharmacovigilance approach. This term encompasses the broad scope of spontaneously reported events in our analysis, where causal attribution may not always be definitively established. We will standardize the use of "ADE" and its abbreviation throughout the text to ensure consistency, reflecting the nature of observational pharmacovigilance data and maintaining alignment with FDA’s adverse event reporting conventions.

**3) Methods: The following text belongs in the data analysis, not data collection, section: “The data was analyzed using R software (Version 4.3.1, https://www.r-project.org/).**

***Response:*** Thank you for your valuable suggestion. We have carefully considered your comment and agree that the sentence "The data was analyzed using R software (Version 4.3.1, https://www.r-project.org/)" is more appropriately placed in the data analysis section rather than the data collection section. These revisions have been incorporated into the revised **Methods section 2.2 (paragraph 2)**.

4) **Results: The ROR, PRR, EBGM, and IC values appear very high. “Additionally, ADEs such as male endometriosis (n = 7; ROR 13,615.84, PRR 13,612.3, IC 10.73, EBGM 1,702.41), 5-alpha-reductase deficiency (n = 5; ROR 1,620.81, PRR 1,620.51, IC 9.79, EBGM 884.37), congenital bulbospinal muscular atrophy (n = 6; ROR 402.42, PRR 402.33, IC 8.38, EBGM 333.53), and double-hit lymphoma (n = 5; ROR 243.12, PRR 243.08, IC 7.76, EBGM 216.18).” It may be useful to expand your methods to describe how each of these measures are calculated.**

***Response:*** Thank you for your feedback. The high ROR, PRR, EBGM, and IC values for rare ADEs stem from their extreme rarity in the FAERS database, where events like male endometriosis (n=7) occur in very few spironolactone reports and scarcely in non-spironolactone reports, leading to disproportionately large ratio calculations. These values are derived using a 2×2 contingency table (a=spironolactone+ADE, b=spironolactone-ADE, c=non-spironolactone+ADE, d=non-spironolactone-ADE), with methods like ROR = (a/c)/(b/d) and PRR = [a/(a+b)]/[c/(c+d)], each requiring thresholds (e.g., ROR ≥3, 95% CI >1) for significance. Despite high values, multi-method validation (BCPNN, EBGM) confirms signals are statistically significant and not due to random chance.

We sincerely thank you again for your thoughtful comments and constructive suggestions. We hope that the revised manuscript meets the requirements and expectations. Please do not hesitate to contact us should any further clarification be needed.

With best regards,

Lixian Li

[lilixian007@sina.com](mailto:lilixian007@sina.com)
